# Landmark precision and reliability and accuracy of linear distances estimated by using 3D computed micro-tomography and the open-source TINA Manual Landmarking Tool software

**DOI:** 10.1186/s12983-015-0101-5

**Published:** 2015-06-03

**Authors:** Monique Nouailhetas Simon, Gabriel Marroig

**Affiliations:** Departamento de Genética e Biologia Evolutiva, Universidade de São Paulo, Rua do Matão, 277, 05508-090 São Paulo-SP, Brazil

**Keywords:** Bone density, Distance repeatability, Geometric morphometrics, Traditional morphometry, Toad skulls

## Abstract

**Introduction:**

The wider availability of non-destructive and high-resolution methods, such as micro-computed tomography (micro-CT), has prompted its use in anatomical and morphometric studies. Yet, because of the actual scanning procedure and the processing of CT data by software that renders 3D surfaces or volumes, systematic errors might be introduced in placing landmarks as well as in estimating linear distances. Here we assess landmark precision and measurement reliability and accuracy of using micro-CT images of toad skulls and the TINA Manual Landmarking Tool software to place 20 landmarks and extract 24 linear distances. Landmark precision and linear distances calculated from 3D images were compared to the same landmarks and distances obtained with a 3D digitizer in the same skulls. We also compared landmarks and linear distances in 3D images of the same individuals scanned with distinct filters, since we detected variation in bone thickness or density among the individuals used.

**Results:**

We show that landmark precision is higher for micro-CT than for the 3D digitizer. Distance reliability was very high within-methods, but decreased in 20 % when 3D digitizer and micro-CT data were joined together. Still, we did not find any systematic bias in estimating linear distances with the micro-CT data and the between-methods errors were similar for all distances (around 0.25 mm). Absolute errors correspond to about 6.5 % of the distance’s means for micro-CT resolutions and 3D digitizer comparisons, and to 3 % for the filter type analysis.

**Conclusions:**

We conclude that using micro-CT data for morphometric analysis results in acceptable landmark precision and similar estimates of most linear distances compared to 3D digitizer, although some distances are more prone to discrepancies between-methods. Yet, caution in relation to the scale of the measurements needs to be taken, since the proportional between-method error is higher for smaller distances. Scanning with distinct filters does not introduce a high level of error and is recommended when individuals differ in bone density.

**Electronic supplementary material:**

The online version of this article (doi:10.1186/s12983-015-0101-5) contains supplementary material, which is available to authorized users.

## Introduction

The increasing use of non-destructive and high-resolution data acquisition methods, such as micro-computed tomography (micro-CT), have provided researchers with the opportunity to study the anatomy and morphology of organisms with more detail and at a wider phylogenetic spectrum (e.g. [1–5]). Accordingly, 3D image processing software has been developed (e.g. OsiriX: [6], Amira: www.amira.com), with some designed to place 3D landmarks for shape or morphometric analysis (e.g. TINA Manual Landmarking Tool: [7]). However, there is no guarantee that the scanning procedure and the software used to process CT data, by creating 3D surface or 3D volume renderings, do not introduce systematic errors in the data [8, 9]. In addition, the landmark positioning process in the 3D images might also introduce systematic and random errors in the estimation of linear distances (measurements). Thus, the precision of placing landmarks in 3D images with software, as well as the accuracy and reliability of the distances taken by the use of CT data must be validated [8–10].

In this study, we evaluated the precision of placing the same landmarks in the same individuals of a toad species with two distinct methods: (1) the real skulls and a 3D digitizer to place the landmarks, and (2) 3D images of these skulls, obtained by micro-CT scans at two distinct resolutions, and software to place the landmarks (TINA Manual Landmarking Tool ([7]; hereafter called TINA-Landmark). The 3D digitizer is an articulated arm that creates a 3D coordinate system in which any point of an object can be identified in relation to a reference point. TINA-Landmark is recently developed open-source software created to enhance the precision of landmark positioning in 3D images by using volume rendering instead of surface rendering and by showing the cross-section images connected to the 3D volume [7]).We chose to compare the landmarks obtained from the 3D images with the ones obtained by the 3D digitizer because, for the latter, the landmarks are taken in the actual skulls, with no processing of data, not the case for the construction of 3D images. Also, in zoological studies, several authors measure the specimens with 3D digitizers (e.g. [11–14], just to cite a few), being a widely accepted technique in the morphometry field.

In addition to comparing the landmarks between the two methods, it is also of interest to evaluate the consequence of potential biases in the estimation of linear distances extracted from the landmarks. Although measurement error is intrinsic to the landmarks and independent of the linear distances computed from them, the proportion of error varies with the distance length. That is, if the error in placing landmarks is the same for all landmarks, the proportion of error will be higher for smaller distances than for longer distances [15]. On the other hand, if the error in placing landmarks varies with landmark type or position in the material, linear distances extracted from these more variable landmarks are expected to have greater error when comparing distinct methods, although an association between distance length and error proportion will still exist. Therefore, we also calculated reliability and accuracy of linear distances extracted from the landmarks placed with the 3D digitizer and in the 3D images.

**Fig. 1:**
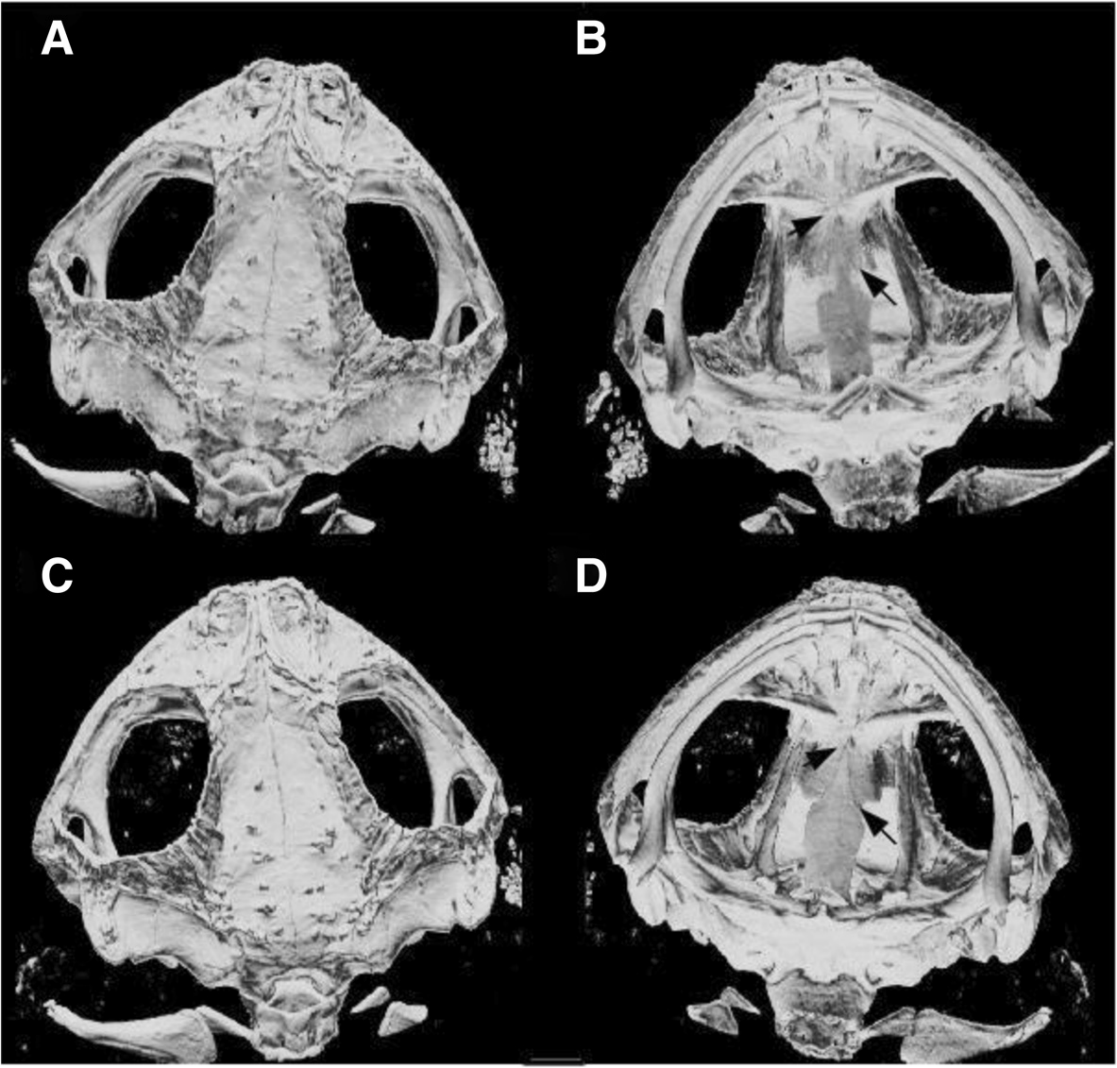
Skull 3D images of a *Rhinella pygmaea* specimen scanned with distinct filters. Several *R. pygmaea* specimens presented transparency in some bones, such as the squamosal, sphenethmoid and the parasphenoid bones, making the precise determination of sutures between these bones difficult (indicated by the arrows). Scanning with a thinner filter corrects for this problem as can be seen in **c** and **d**. **a** Dorsal view of a skull scanned with an aluminum filter 1.0 mm (AL1.0); **b)** Ventral view of the same skull scanned with AL1.0; **c)** Dorsal view of the same skull scanned with an aluminum filter 0.5 mm (AL0.5); **d)** Ventral view of the same skull scanned with AL0.5

Finally, we also compared the same toad specimens scanned with two distinct filters, since we discovered that some individuals differ in bone thickness and/or density. Filters are thin sheets of metal set in front of the material being scanned and can have different thickness. Varying the filter thickness has an effect on the mean X-ray energy irradiating on the material being scanned. For thinner bones, a lower X-ray energy is necessary to achieve the best 3D volumes and more precise positioning of the landmarks (see Fig. 1). Thus, changing filter type might be an additional source of error when placing landmarks and taking linear measurements in 3D images. We consider this last comparison quite relevant in zoological studies because other organisms might present the same variation in bone density and scanning with distinct filters will be indispensable.

**Fig. 2:**
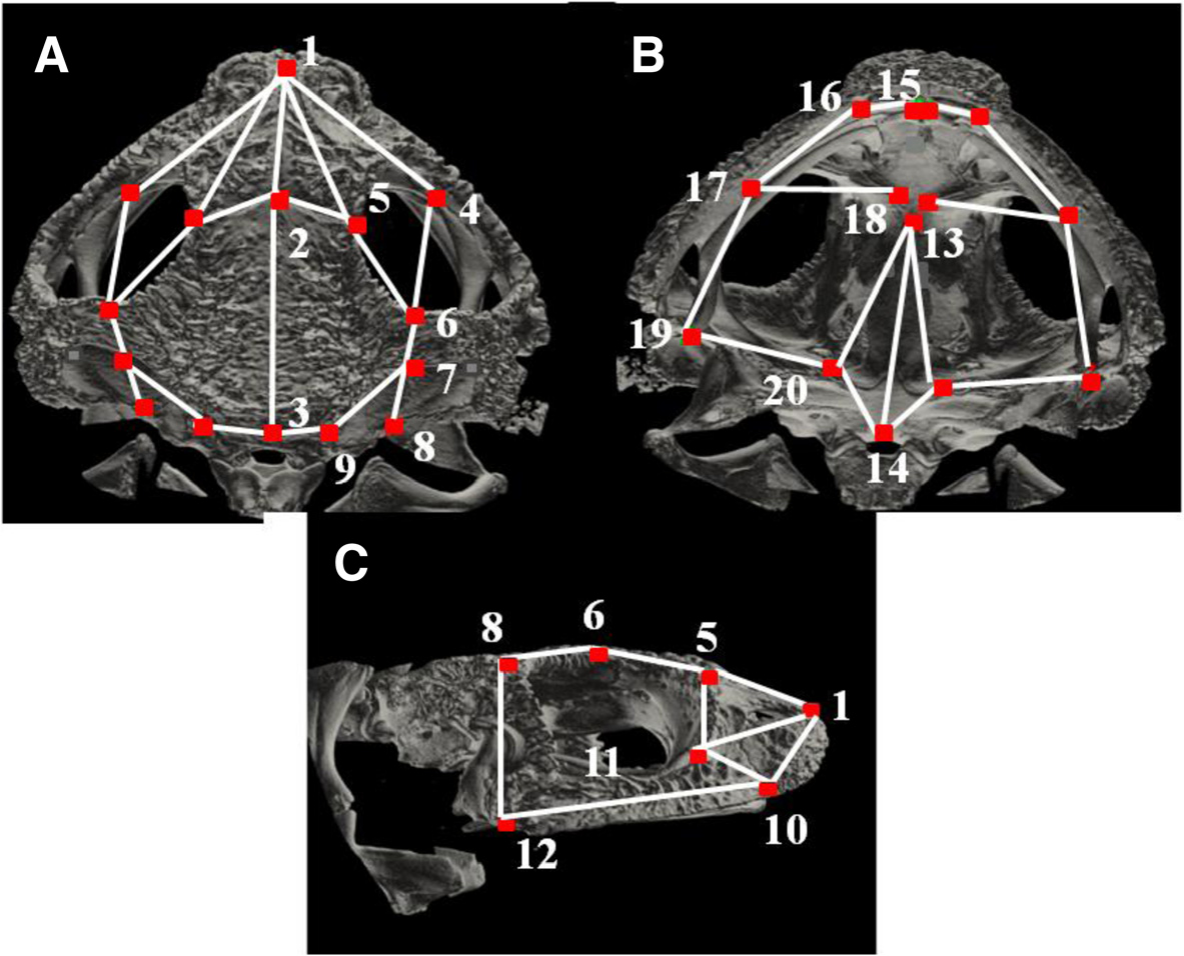
Landmarks and linear distances used in the toad skulls. Numbered landmarks in both sides of the skull are shown as red dots in dorsal (**a**) and ventral (**b**) views, and only landmarks of the right side of the skull are shown in the lateral (**c**) view (descriptions in Table 1). Landmarks were placed in bone sutures or bone processes either with TINA-Landmark software in 3D images or with a 3D digitizer in the real skulls. Linear distances are shown as white lines and represent individual bone dimensions, as shown in Table 2

Although there is no specific theory relating the potential effects on landmarks and on distances when using distinct resolutions, filters and reconstruction algorithms, some expectations based on landmark position and bone thickness can be made. Some of the landmarks that we selected in the toad skulls (Table 1 and Fig. 2) were more difficult to visualize in the 3D images than in the real skulls because of their position (landmarks 4, 10, 11, 17 and 20) or because they were placed at thinner bones (landmarks 6, 7, 8 and 13). Thus, we expect more variation in the positioning of these landmarks among methods, and as a consequence, less reliability and accuracy of the linear distances extracted from them (Table 2 and Fig. 2). We report that placing landmarks in 3D images obtained by micro-CT scanning is more precise than placing the same landmarks with the 3D digitizer. Yet, average differences in linear distances among methods are acceptable and represent a low error proportion in relation to the distances lengths. Scanning with distinct resolutions and distinct filters do not introduce high errors.

**Table 1:** Landmark descriptions in the toad skulls. Landmarks are intersections between bone sutures (type I landmarks, 16 in total) or tip of bones (type II landmarks: numbers 1, 14, 15 and 19). Five landmarks are in the medial line and the remaining landmarks are present in both sides of the skull. The landmarks are spread in all three views of the skull: dorsal, lateral and ventral (see Fig. 1). We placed all 20 landmarks with all the methods twice in each individual

Landmarks	Description	Position	View
1	Anterior tip of nasal bone	midline	dorsal
2	Nasal and frontoparietal suture	midline	dorsal
3	Posterior tip of frontoparietal suture	midline	dorsal
4	Nasal and maxillary suture	right, left	dorsal
5	Nasal and frontoparietal lateral suture	right, left	dorsal
6	Frontoparietal and squamosal suture	right, left	dorsal
7	Frontoparietal, squamosal and occipital suture	right, left	dorsal
8	Squamosal and occipital suture	right, left	dorsal
9	Frontoparietal and occipital suture	right, left	dorsal
10	Prenasal and maxillary lateral suture	right, left	lateral
11	Nasal and maxillary lateral suture	right, left	lateral
12	Squamosal and maxillary suture	right, left	lateral
13	Sphenethmoid and parasphenoid suture	midline	ventral
14	Posterior tip of parasphenoid corpus	midline	ventral
15	Anterior tip of premaxillary bone	right, left	ventral
16	Premaxillary and maxillary suture	right, left	ventral
17	Pterygoid and maxillary suture	right, left	ventral
18	Neopalatine and sphenethmoid suture	right, left	ventral
19	Tip of pterygoid process	right, left	ventral
20	Pterygoid and parasophenoid suture	right, left	ventral

**Table 2:** Linear distances determined in the toad skulls. In total, we determined 24 linear distances representing individual dimensions of the bones (or the orbit) in the toads’ skulls. Distances are spread through the skull in three views (dorsal, ventral and lateral; see Fig. 1). We positioned landmarks in both sides of the skull and averaged the distances from both sides

Distances	Landmarks	Bones
1	1-2	nasal
2	2-3	frontoparietal
3	1-4	nasal
4	1-5	nasal
5	2-5	frontoparietal
6	5-6	frontoparietal
7	4-6	orbit
8	6-8	squamosal
9	7-9	occipital
10	3-9	frontoparietal
11	1-10	prenasal
12	1-11	nasal
13	10-11	nasal
14	5-11	nasal
15	10-12	maxilla
16	8-12	squamosal
17	13-14	parasphenoid
18	13-20	parasphenoid
19	15-16	premaxilla
20	16-17	nasal
21	17-18	neopalatine
22	17-19	pterygoid
23	19-20	pterygoid
24	14-20	parasphenoid

## Results and discussion

### Landmark precision with distinct methods

We compared landmark precision between-methods by calculating the mean distance of each landmark in the individuals from the same landmarks of a mean shape estimated with a slightly modified General Procrustes Analysis, for all methods (see Methods section). Mean individual landmark distances from the mean sample landmarks were higher when using the 3D digitizer (DIG) to place the landmarks than when using the 3D images, regardless of the resolutions (medium [MED] or high [HIGH]). This result holds for the shape space (without scale, Table A1 in Additional file 1), as well as for retaining landmark scale information in mm (after multiplying the Procrustes configuration by mean centroid size, Table 3), indicating that placing landmarks in 3D images obtained by the micro-CT is more precise. Even though the precisions of both equipments used in this study are similar (3D digitizer: 0.01 mm; micro-CT resolutions MED: 0.018 mm and HIGH: 0.009 mm), this result may be due to the fact that the 3D images of the skulls are much bigger in the screen than the real ones (around tenfold increase), facilitating visualization of several bone structures. The range of deviations of individual landmarks from the mean shape landmarks for all methods (DIG: 0.27 to 0.52 mm; HIGH: 0.14 to 0.33 mm and MED: 0.16 to 0.34 mm) are comparable to the error found by Richtsmeier et al. (1995) [16] when placing the same landmarks in two different 3D images of the same individual (scanned twice with a 1.5 mm slice thickness resolution), which was 0.15 to 0.48 mm. Thus, the precision of both methods in placing landmarks is acceptable for morphometric studies, at least for distances as large as the ones obtained in this study.

**Table 3:** Within-methods mean individual landmark distances in mm

Mean in mm									
Landmarks	DIG	HIGH	MED	AL1.0	AL0.5	S2	S2/D2	S3	S3/D2
1	0.299	0.227	0.219	0.267	0.221	0.234	0.227	0.220	0.239
2	0.284	0.212	0.242	0.165	0.168	0.236	0.234	0.215	0.246
**3**	**0.357**	0.188	0.182	0.243	0.222	0.182	0.191	0.184	0.172
4	0.290	0.203	0.205	0.208	0.238	0.270	0.261	0.270	0.268
5	0.363	0.245	0.232	0.259	**0.422**	0.230	0.239	0.248	0.227
**6**	**0.365**	0.187	0.203	0.256	0.296	0.210	0.191	0.199	0.213
7	**0.345**	0.177	0.179	0.170	0.149	0.163	0.167	0.171	0.160
8	0.338	0.323	0.266	0.290	0.374	0.289	0.227	0.300	0.292
**9**	**0.473**	0.215	0.219	0.271	0.289	0.227	0.207	0.233	0.210
**10**	**0.380**	0.182	0.179	0.190	0.209	0.174	0.171	0.181	0.182
**11**	**0.303**	0.182	0.191	0.180	0.160	0.191	0.183	0.181	0.148
**12**	**0.435**	0.282	0.301	0.262	0.254	0.296	0.285	0.287	0.266
13	0.341	0.275	0.298	0.337	0.383	0.267	0.259	0.271	0.275
**14**	**0.500**	0.226	0.205	0.192	0.183	0.195	0.202	0.220	0.219
**15**	**0.342**	0.141	0.152	0.166	0.200	0.147	0.140	0.144	0.137
**16**	**0.252**	0.137	0.140	0.161	0.179	0.167	0.128	0.147	0.144
17	0.266	0.173	0.201	0.218	0.171	0.189	0.186	0.185	0.196
18	0.355	0.226	0.232	0.199	0.240	0.224	0.257	0.236	0.224
**19**	**0.516**	0.216	0.252	0.282	0.286	0.220	0.214	0.209	0.214
**20**	**0.481**	0.196	0.175	0.222	0.226	0.192	0.199	0.191	0.187

The table shows the mean deviation of individual landmarks from the mean landmark. We used Generalized Procrustes Analysis (GPA) to superimpose individual landmarks, but we avoided the spread of variation from any one landmark to the others (see text). To get back to a scale in mm, all the mean deviation values were multiplied by the mean centroid size of the correspondent sample. Values in bold are the highest deviations for DIG compared to MED and HIGH or for AL0.5 compared to AL1.0

The two landmarks with highest mean deviations for DIG (14: posterior tip of parasphenoid process and 19: posterior tip of pterygoid process) were not expected to have higher error, however they both are type II landmarks, suggesting that locating the exact maximum bone curvature was more difficult than locating bone sutures (type I landmarks). The higher error for type II landmarks compared to type I landmarks is expected when working with the real skulls [17]. Landmarks 3, 5, 6, 7, 9, 10, 11, 12, 15, 16 and 20 presented higher discrepancies in the landmarks for DIG when compared to the micro-CT resolutions (boldface in Table 3). For the landmarks expected to have higher error in the 3D images because of their positions in the skull or because they were located in thinner bones, only landmarks 4, 8 and 12 corresponded to the expectation. All of them are type I landmarks, agreeing with the findings of other authors working with 3D images of more error in placing landmarks at some particular bone sutures [18, 19].

MED and HIGH presented very similar results, indicating that there is no difference in switching the resolutions to visualize the landmarks. The fact that we had to down-sample the HIGH data by a factor of two to load the cross-section sequences in TINA-Landmark probably did not interfere in landmark precision, since switching the compacting factors with the MED data also produced similar results (comparisons between stride = 2 [S2] and stride = 2 and down-sample = 2 [S2/D2]; and stride = 3 [S3] and stride = 3 and down-sample = 2 [S3/D2]; Table 3). This result is consistent with the fact that the cross-sections are maintained in the original resolution even when down-sampling the data (which affects only the resolution of the 3D volume), being possible to refine the landmarks positions in them. Finally, for the filter type analysis, landmarks 6, 8 and 13 presented higher mean individual landmark distances for aluminum 0.5 mm (AL0.5) than for aluminum 1.0 mm (AL1.0), coinciding with the ones expected as more variable for being located at thinner bones. The only exception was landmark 5, with high deviation for AL 0.5. This landmark is located in the nasal and frontoparietal lateral suture, coinciding with a neural crest. It is possible that the presence of the crest caused higher error in the positioning of the landmark.

### Linear distances reliability and accuracy

**Fig. 3:**
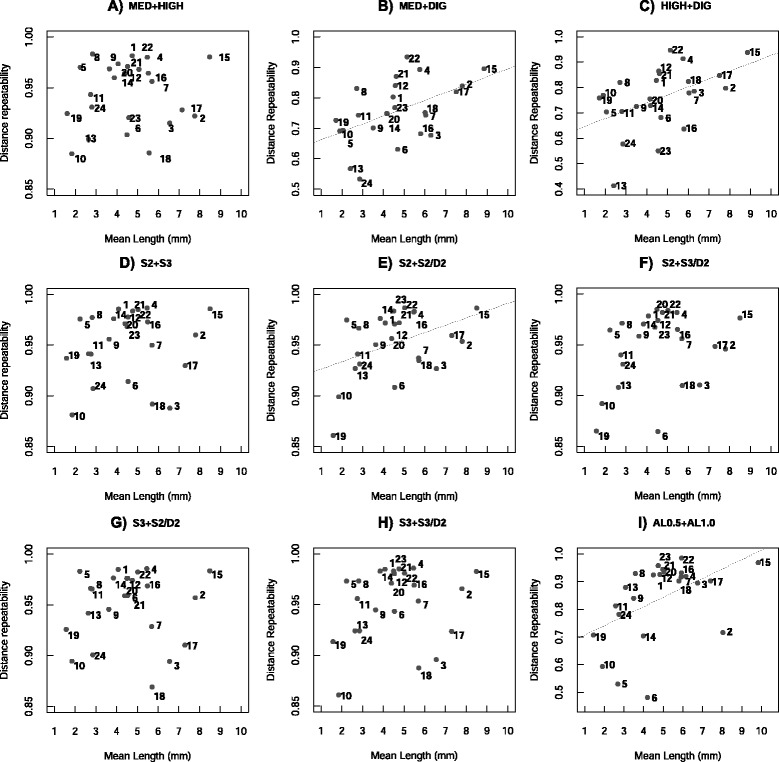
Between-method distance repeatability in relation to distance mean length. Individual toad skulls were each measured twice with different methods: using a 3D digitizer (DIG); using CT scans at medium (MED) or high (HIGH) resolutions; CT scans at MED loaded in TINA-Landmark with distinct values of the compacting factors stride and down-sample (S2, S3, S2/D2 and S3/D2); and CT scans at MED with 0.5 mm aluminum filter (AL0.5) or 1.0 mm aluminum filter (AL1.0). Data from different methods were joined together and distance repeatabilities calculated, representing the reliability in placing landmarks with the different methods. The dashed lines indicate significant correlations between the variables. Numbers 1–24 correspond to the distances as described in Table 2. **a)** MED + HIGH; **b)** DIG + MED; **c)** DIG + HIGH; **d)** S2 + S3; **e)** S2 + S2/D2; **f)** S2 + S3/D2; **g)** S3 + S2/D2; **h)** S3 + S3/D2 and **i)** AL0.5 + AL1.0

The reliability of the linear distances was obtained by using distance repeatability, which indicates the reliability of multiple measurements in the same individuals. It describes the proportion of variance due to differences among individuals (between-group variance) in relation to the residual variance plus the between-group variance [20]. Within-method mean distance repeatabilities (i.e. considering only replicates measured with the same method) were very high, above 0.9 for all methods, as can be seen in Table 4 for micro-CT resolutions and 3D digitizer; and in Table 5 for image compacting factors and distinct filters. This result shows that measuring procedure is very reliable inside each method. However, between-methods mean distance repeatability is considerably lower (20 % lower) when replicates of MED or HIGH were joined with replicates of DIG, but not when replicates of distinct micro-CT resolutions were joined together (Table 4). When replicates of AL0.5 and AL1.0 were joined together, the mean repeatability also reduced (Table 5), yet the drop was less steep (13 % lower) than MED + DIG and HIGH + DIG. For the image compacting factors there was no drop in mean distance repeatability when replicates of different values of the compacting factors were considered together (Table 5). Looking at repeatabilities separately for each distance in the between-methods analysis (Fig. 3b,c), it can be noticed that distances 3, 5, 6, 9, 10, 11, 13, 14, 16, 23 and 24 all have low repeatabilities (below 0.8) and are composed of landmarks detected with higher mean deviations for DIG (landmarks 3, 5, 8, 9, 10, 11, 12 and 19). These distances comprise small as well as large distances, showing that the error is independent of distance length and related to the quality of the landmark. Although we did not make expectations of higher error for landmarks placed with DIG, the landmarks with higher deviations had as a consequence higher discrepancies in the between-methods repeatabilities. However, the distances 17 and 22, composed of the landmarks with the highest error for DIG (14 and 19), did not present low repeatabilities. This is a consequence of their longer mean lengths (17: 7.14 mm and 22: 5.06 mm) and a smaller proportional error as a consequence. On the other hand, distances 5, 10, 11, 13 and 24 are small distances (less than 3.0 mm of length) composed of highly discrepant landmarks, and therefore, have a larger proportional error. These differences in proportional error depending on the mean distance length is shown by a significant positive association between mean distance repeatability and mean distance length for the joined data sets MED + DIG and HIGH + DIG (r = 0.45, d.f. = 22, P = 0.02 and r = 0.44, d.f. = 22, P = 0.03, respectively; Fig. 3b,c), as well as for S2 + S2/D2 and AL0.5 + AL1.0 (r = 0.44, d.f. = 24, P = 0.03 and r = 0.52, d.f. = 24, P = 0.007, respectively; Fig. 3e,i). Although a significant relation between repeatability and distance length exists, practically all distances composed of landmarks with higher differences between-methods presented lower repeatability when compared to within-method repeatability.

**Table 4:** Within and between methods mean distance repeatabilities and mean raw and absolute differences for the 3D digitizer (DIG) versus micro-CT resolutions (MED or HIGH) comparison. Each individual was measured twice by each of the three methods and mean ± s.d. distance repeatabilities were calculated for within (considering only replicates of the same individual measured with the same method) and between-methods (considering the same individual measured with different methods). Within and between methods calculations were also done for raw and absolute differences between linear distances (mean ± s.d.). The last line of the table shows the mean between-method percentage error in relation to distance means

Mean distance repeatabilities			
**Within**	**DIG**	**MED**	**HIGH**
	0.94 ± 0.07	0.97 ± 0.02	0.98 ± 0.01
**Between**	**MED + HIGH**	**DIG + MED**	**DIG + HIGH**
	0.95 ± 0.03	0.76 ± 0.10	0.76 ± 0.12
**Mean differences (mm)**			
Within	**DIG**	**MED**	**HIGH**
Raw	0.01 ± 0.04	−0.002 ± 0.02	−0.003 ± 0.02
Absolute	0.09 ± 0.03	0.07 ± 0.02	0.06 ± 0.02
Between	**MED-HIGH**	**DIG-MED**	**DIG-HIGH**
Raw	−0.03 ± 0.14	−0.02 ± 0.28	0.01 ± 0.28
Absolute	0.11 ± 0.05	0.27 ± 0.1	0.26 ± 0.1
% of mean	2.5 ± 1.0	6.5 ± 2.3	6.4 ± 3.0

**Table 5:** Within and between methods mean distance repeatabilities and mean raw and absolute differences for the image compacting factor comparison and the filter type comparison

Mean distance repeatabilities						
**Within**	**S2**	**S3**	**S2/D2**	**S3/D2**	**AL0.5**	**AL1.0**
	0.97 ± 0.02	0.98 ± 0.03	0.97 ± 0.02	0.97 ± 0.02	0.95 ± 0.04	0.93 ± 0.10
**Between**	**S2 + S3**	**S2 + S2/D2**	**S2 + S3/D2**	**S3 + S2/D2**	**S3 + S3/D2**	**AL0.5 ± AL1.0**
	0.95 ± 0.03	0.95 ± 0.03	0.95 ± 0.04	0.95 ± 0.03	0.95 ± 0.04	0.83 ± 0.16
**Mean differences (mm)**						
**Within**	**S2**	**S3**	**S2/D2**	**S3/D2**	**AL0.5**	**AL1.0**
**Raw**	−0.002 ± 0.02	−0.003 ± 0.01	0.008 ± 0.02	0.006 ± 0.02	0.0 ± 0.02	0.003 ± 0.03
**Absolute**	0.7 ± 0.02	0.06 ± 0.02	0.07 ± 0.02	0.07 ± 0.02	0.07 ± 0.02	0.07 ± 0.03
**Between**	**S2-S3**	**S2-S2/D2**	**S2-S3/D2**	**S3-S2/D2**	**S3-S3/D2**	**AL0.5-AL1.0**
**Raw**	−0.01 ± 0.13	0.0 ± 0.12	0.0 ± 0.13	0.02 ± 0.13	0.02 ± 0.13	−0.03 ± 0.21
**Absolute**	0.10 ± 0.04	0.09 ± 0.04	0.10 ± 0.04	0.10 ± 0.06	0.10 ± 0.05	0.14 ± 0.05
**% of mean**	2.3 ± 0.9	2.3 ± 1.1	2.4 ± 1.0	2.4 ± 1.1	2.3 ± 1.0	3.4 ± 2.0

The four distinct values of the image compacting factors stride and down- sample were: (S2): stride = 2.0; (S3): stride = 3.0; (S2/D2): stride = 2.0 and down-sample = 2.0 and (S3/D2): stride = 2.0 and down-sample = 3.0. The two different scanning filters were: aluminum 0.5 mm (AL0.5) and aluminum 1.0 mm (AL1.0). Within and between methods mean ± s.d. distance repeatabilities and mean ± s.d. raw and absolute difference were calculated, as well as mean percentage error in relation to the distances means of the between methods error

Additionally to the distance repeatability analysis, we also compared mean raw and absolute differences in the distances within and between methods. The mean raw differences within and between methods were close to zero for all data sets, being sometimes positive and sometimes negative (Tables 4 and 5), indicating that in average there is no consistent bias when measuring the same individual two times or measuring the same individuals with different methods. Yet, when looking at the raw differences between MED-DIG and HIGH-DIG for the distances separately, we can see that some distances have higher differences, ranging from −0.38 mm to 0.36 mm (Table A2 in the Additional file 1). These distances correspond to different bones in the toad skulls, indicating that the deviations in the relative landmark positions are not localized in a few bones. We can see that the higher differences (above 0.17 mm in magnitude, the highest difference found for MED-HIGH, values in boldface in Table A2) have approximately the same magnitude in mm for small and long distances. For instance, small distances 5, 8 and 13 (distance means below 3.0 mm) have a mean between-methods difference of 0.23 mm, while long distances have a mean of 0.26 mm. However, as mentioned above, the same magnitude of between-methods error for small and long distances results in a higher proportion of error in relation to the distances means when considering the smaller ones (Table A3 in Additional file 1). When looking at the between-methods mean percentage error, the error in relation to the distances mean lengths, we can see that the cases around 10 % correspond to three small distances: 5 (frontoparietal bone), 13 (nasal bone) and 25 (parasphenoid bone), and just one longer distance: 16 (squamosal bone), which is composed of two landmarks detected with high deviations for DIG (landmarks 8 and 12). The magnitudes of differences that we found for DIG and the micro-CT resolutions are similar to differences reported by other authors when comparing measurements taken with CT data and digital calipers [18, 21], although the last authors did find a systematic bias in the CT data (all distances under-estimated). Yet, when comparing our results with other authors that measured the same specimens with a 3D digitizer (Polhemus 3Space) and CT [15, 19], our error between-methods is much smaller (around five to ten times smaller). This is probably due to the fact that we have used a much higher resolution than these authors in both the micro-CT as well as the digitizer since current equipments are an order of magnitude more accurate than 20 years ago.

**Fig. 4:**
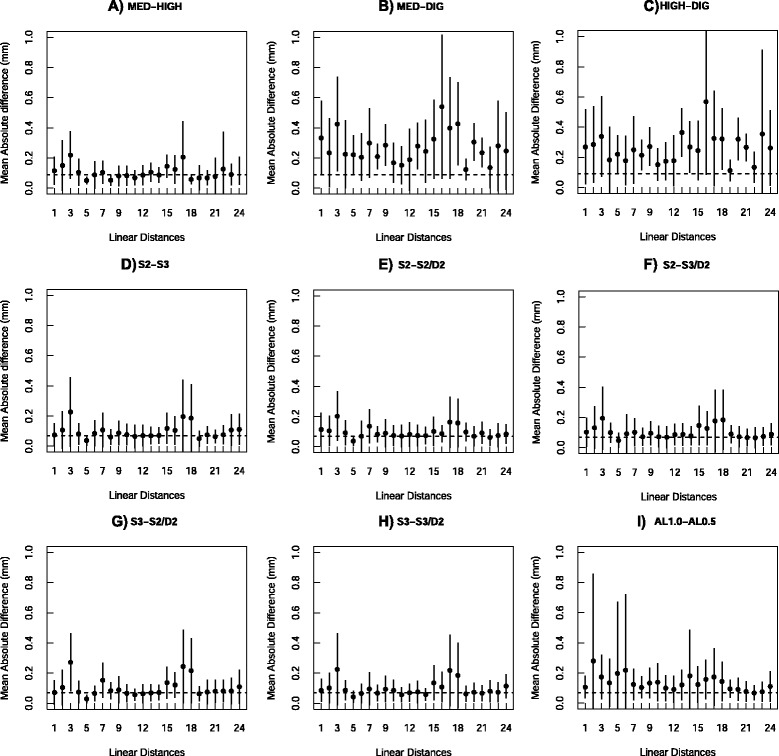
Between-method mean absolute differences in linear distances. Using the same data sets as described in Fig. 3, absolute differences in mm between replicates of the same individuals measured with different methods were calculated and averaged across individuals. Circles represent means and vertical lines are the s.d. Dashed lines indicate the mean within-method absolute differences. Numbers 1–24 correspond to the distances as described in Table 2. **a)** MED + HIGH; **b)** DIG + MED; **c)** DIG + HIGH; **d)** S2 + S3; **e)** S2 + S2/D2; **f)** S2 + S3/D2; **g)** S3 + S2/D2; **h)** S3 + S3/D2 and **i)** AL0.5 + AL1.0

The only distances estimated with more error when scanning at different resolutions were distances17 and 19 (0.17 mm of difference), both distances in the parasphenoid bone, one of the thinner bones in the toad skull. It is possible that in this case scanning with HIGH enhanced the visualization of the sutures in the parasphenoid bone compared to MED. Scanning with HIGH instead of with MED increases the scanning time per skull over 4 times (20 min versus 1.5 h), so the advantage in enhancing the accuracy of some distances by using higher resolution depends on the question being asked and the time available. The between-methods absolute differences for each distance (Fig. 4) also indicate that some distances had higher magnitudes of difference. The worst case was for distance 16 (squamosal bone), the only long distance that presented percentage error around 10 % and an error around 0.5 mm. This magnitude of error is very close to what we considered a gross error when measuring the individuals inside each method. When looking at the distinct filter results (AL1.0-AL0.5), we can see that both the raw and absolute differences between individuals scanned with distinct filters are much lower than differences between micro-CT resolutions and 3D digitizer. Similarly, in table A3 we can notice that the highest mean percentage error for filter type comparisons was around 7 %, and all related to small distances.

## Conclusions

In order to use 3D images of organisms in morphometric studies, one needs to know if the relative positions among landmarks are kept the same for different scanning procedures or rendering algorithms. By comparing landmark precision and linear distances in toad skulls scanned with two resolutions and measured with the software TINA-Landmark, with the same skulls measured with a 3D digitizer, we conclude that the degree of discrepancy is acceptable in general, although several distances had between-methods discrepancies above 5 %. Yet, we must consider that 30 % of the distances are below 3.0 mm in length, being quite small distances, thus for several other bigger organisms the error proportion will probably be lower than what we report. Attention needs to be paid in relation to the scale of the distances, as smaller distances might be estimated with proportionally more error. Using distinct micro-CT resolutions, distinct compacting factors for loading 2D cross-section sequences in TINA-Landmark, as well as using distinct filters to scan individuals that differ in bone density do not introduce high errors. We recommend the switching of filters when individuals to be compared differ in bone density, at least with the resolutions that we have tested.

## Methods

### Species and scanning procedures

For the micro-CT and 3D digitizer comparisons, as well as for the 3D image compacting factor analysis, we used 20 adult individuals of the toad species *Rhinella granulosa*, collected in January 2012 at a site near the municipality of Angicos (5° 39′ S, 36° 36′W), in the state of Rio Grande do Norte, NE Brazil. The toads were sacrificed by peritoneal application of an anesthetic in excess and then were fixed in 70 % alcohol. We scanned the toad skulls with an X-ray micro-CT system (SkyScan 1176, Konitch, Belgium) placed at the Instituto de Biociências, Universidade de São Paulo. All individuals were scanned with a 1.0 mm AL filter at two different resolutions: medium (**MED**: 18 μm, 70 kV, 356 μA) and high (**HIGH**: 9 μm, 65 kV, 380 μA). These resolutions correspond to different voxel sizes, the smallest volume unit in the 3D volumes: 3.85 10^−6^ mm^3^ for MED and 1.92 10^−6^ mm^3^ for HIGH, which are much smaller than conventional CTs (voxel sizes ranging from 0.1 to 5 mm^3^). Before scanning, toads were wrapped with Parafilm to avoid too much alcohol evaporation and consequent dehydration, which could lead to blurred images by sample contraction. The scanning time per skull for MED was 20 min. and for HIGH was 1.5 h. After scanning, the skulls were reconstructed using NRecon software (SkyScan, Konitch, Belgium). In this process, 2D projection images are reconstructed to cross-section images by use of a mathematical algorithm (Feldkamp). The first step in the reconstruction process was to choose the lower and upper limits of the threshold for the linear attenuation coefficient (AC). The AC measures how much the intensity of the X-ray beam is reduced as it passes through the material being scanned, and is related to the density of the materials. Based on the histograms of soft tissue and bone density of the skulls, we chose an AC threshold of 0.0 and 0.05 for all specimens scanned. Afterwards, we applied different types of corrections in the reconstruction process to soften some undesirable effects in the images: post-alignment = −5.0 to 2.0; ring artifact reduction = 2.0 to 4.0; beam-hardening correction = 30 % and smoothing = 2.0. These corrections are important to avoid blurring and artifacts in the 3D images and for all corrections the values used were small compared to maximum values. Therefore, we do not expect these corrections to interfere in landmark visualization, but instead to improve its identification. The sequences of cross-section images varied from 400 to 600 images for MED (500 MB to 1 GB per skull file size) and from 800 to 1,200 images for HIGH (1 to 5 GB per skull file size) and were all in BMP extension.

For the filter type analysis, we used 20 adult museum specimens of *Rhinella pygmaea* (MZUSP, São Paulo, Brazil). This species was chosen because several specimens presented too much transparency or even holes in their skulls, especially in the squamosal, sphenethmoid and parasphenoid bones, preventing the placement of some landmarks and also suggesting that there were differences in bone density among individuals (Fig. 1). We scanned the 20 toads at medium resolution using two distinct filters: 1.0 mm AL (**AL1.0**: 70 kV, 356 μA) and 0.5 mm AL (**AL0.5**: 50 kV, 500 μA). Filters are thin metal sheets that are set in front of the X-ray source and can have different thickness. Filtration retains a part of the low energy photons of the X-ray, thus increasing mean X-ray energy. The reduction in AL filter thickness from 1.0 mm to 0.5 mm results in a lower mean energy of the X-ray because photons with lower energy traverse the filter and achieve the thinner bones, being retained on them [22]. The individuals used in this analysis presented low transparency and no holes since the objective was to evaluate whether the use of distinct filters changed the landmarks positions and the linear distances obtained in the same individuals. In these toads all landmarks could be placed, which would not be the case if we had used specimens with holes or high transparency. CT system, scanning and reconstruction procedures were the same as described above.

### Landmarking procedure and linear distances in 3D images

We placed 20 landmarks at bone sutures (type I landmarks) or bone processes (type II landmarks; 35 landmarks if counting both sides of the skull, left and right) in all views (dorsal, ventral and lateral; Table 1 and Fig. 1) using TINA-Landmark software [7]. This software provides different views of the data: a 3D volume of the whole skull and three 2D views of the orthogonal cross-sections (axial, sagittal and transversal) throughout the image sequence, enabling landmarks to be precisely placed given that the views are linked (*i.e*. when setting a landmark in the 3D volume, it also appears in all 2D views allowing a refinement of the landmark position). The skull’s cross-section sequences were loaded in TINA-Landmark by using the “Sequence Tool” after the BMP files were converted to DICOM files. The Sequence Tool presents two types of image compacting factors for loading heavy files: “Stride”, which reduces the sequence in the inter-slice direction (*i.e*., stride = 2.0 and “Stride average” = ON means that an average image will be loaded at every two cross-sections from the sequence, resulting in half of the original size of the sequence file); and “Down-sample”, which reduces the sequence along the *x* and *y* directions (*i.e*., down-samples the size of the pixels; [23]). MED resolution data were loaded using stride = 2.0 whereas HIGH resolution data were loaded using stride = 3.0 and down-sample = 2.0, since they were heavier and failed to load with the same compacting factor than MED data. The down-sampling procedure only affects the visualization of the 3D volume of the skulls, not changing the visualization of the cross-section images (axial, sagittal and transversal views).

Appropriate visualization of 3D volumes and determination of bone threshold value (the average density of soft tissue and bone to guarantee that landmarks are placed exactly where the mouse is pointing in the 3D image) were obtained by following the recommendations contained in the TINA Geometrics Morphometrics Toolkit manual [23]. Finally, after all steps of image adjustments, a landmark list was loaded with the 35 points and each was placed two times (two replicates per individual) in the skull’s images of *R. granulosa* and *R. pygmea* individuals, so we could assess measuring reliability. The corresponding *x*, *y* and *z* landmark coordinates were saved in a TXT file, which was later loaded in the R programming environment [24] where distances were calculated.

We determined 24 linear distances in the skull of the toads, all distances representing individual bone dimensions (Table 2, except distance 7 which corresponds to orbit size). To obtain distances in mm, the coordinates of MED and HIGH were multiplied by 0.01742 and 0.00871, respectively, which are the size of the pixels in mm for each resolution. In the case of the *R. pygmea* specimens that were scanned with different filters, landmark coordinates were multiplied by 0.01742 because they were only scanned at medium resolution. Distances from replicates of the same individual (within each method) were inspected for gross measurement error (difference between replicates above 0.5 mm for most distances, except for distances 5, 8, 10, 11, 13, 17 and 24, which have means smaller than 3.0 mm, and were controlled for error above 0.3 mm), and when detected, the landmarks correspondent were placed again in both replicates and corrected. The reference to consider the magnitude of a gross error was based on the precision of the 3D digitizer, which is 0.01 mm. This procedure was adopted because these gross errors could lead to misleading conclusions about the between-methods analysis, since they are actually referred to gross human error inside each method. In order to analyze the possible effect on the landmarks and distances of switching the compacting factors (stride and downsample) when loading the cross-section sequences, we placed the landmarks in the *R. granulosa* skulls scanned in medium resolution using the following values in TINA-Landmark: (**S2**) stride = 2.0; (**S3**) stride = 3.0; (**S2/D2**) stride = 2.0 and down-sample = 2.0; and (**S3/D2**) stride = 3.0 and down-sample = 2.0. Again, each individual at each of these four compacting values were measured twice and gross measurement error between replicates inside each compacting factor was controlled. Thus, total sample size for the compacting factor analysis was 20 individuals for each of the four situations, and 80 in total.

### Landmarking procedure and linear distances using 3D digitizer

The same *R. granulosa* adults that we scanned with MED and HIGH resolutions were cleaned and had their skin removed. In this process, we lost four of the skulls by crushing, 16 skulls remaining. We placed the same landmarks described above in the cleaned skulls by using a 3D digitizer (Microscribe 3DX, IL). Since the toad skulls are quite small (total length around 20.0 mm), we used a binocular loupe to mark the landmarks with a pencil. Then, we placed the digitizer pen in the graphite marks to digitize the landmarks. We placed landmarks in all individuals twice and gross error in the distances from one replicate to the other was also controlled as described before at the time of landmark digitalization. A TXT file was created for the coordinates obtained from DIG and was loaded in R environment in order to calculate the same distances as MED and HIGH. The landmarks and distances obtained by using DIG were considered as the reference for comparisons.

### Within-method mean individual landmark distances

To assess the differences of placing landmarks in the skulls 3D volumes compared to placing them in the real skulls with the 3D digitizer, we used a superimposition method, the Generalized Procrustes Analysis (GPA), to estimate the sample mean landmarks (mean shape) and to calculate individual distances from the mean landmarks within each method. The GPA sumperimposes landmark configurations of several individuals, scaling them to unit centroid size, and uses least-square estimates for translation and rotation parameters [25]. These transformations of the landmarks are needed because there is no natural coordinate system that is common to all individuals that are digitized and a common shape space is achieved when doing this procedure [26, 27]. However, this transformation process confounds variation at different homologous landmarks, i.e., the most variable landmarks among individuals (non-isotropic variation) have their variation spread across other landmarks [16, 26, 27]. Thus, to avoid this problem in our landmark variation analysis, we followed the idea presented in van der Linde and Houle (2009) [27] and excluded from the GPA one landmark at a time to estimate the sample mean shape and the rotation matrices for the 19 remaining landmarks for each individual. By doing that, we precluded that the variation in the landmark of interest got spread through the other landmarks. Afterwards, we multiplied the excluded landmark of each individual by its corresponding rotation matrix (all landmarks of an individual are rotated in the same angle) to have all landmarks in the same new coordinate system, the so called shape space. The last step was to calculate the individual distance of the landmark of interest from the mean landmark (in the shape space, therefore with no scale). This procedure was repeated 20 times for each method, so that all 20 landmarks were excluded at each time and the distances from the individuals and the mean landmark could be calculated. To compare the different methods, we also calculated the mean individual landmark distance from the mean landmarks. Finally, in order to compare the methods of placing landmarks in a more intuitive scale, we multiplied the mean deviations and SD values by its corresponding mean sample centroid size in mm. Only the medial and left side landmarks were used in this analysis and the GPA was done with the “geomorph” [28] and “shapes” [29] packages in R environment.

### Distance repeatability within and between methods

We calculated repeatability values for all distances in every data subset using the calculations described in [20]. Each individual is a group with two replicates and the between-group variance is the sum of squares of the deviations of the group means from the total mean, whereas the within-group variance corresponds to the sum of squares of the deviations of each replicate from its own group mean. The index is calculated as follows:

$$ r=\frac{s_A^2}{\left({s}^2+{s}_A^2\right)} $$

s^2^_A_ being the variance among groups and s^2^ the residual variance, both calculated from the sum of squares in an ANOVA.

We calculated repeatabilities for MED, HIGH and DIG separately, and also for joined data sets, (MED + HIGH), (MED + DIG), (HIGH + DIG), with the three between-method data sets made up of four replicates per individual. In the same manner, we calculated repeatability for replicates of the different values of stride and down-sample, S2, S3, S2/D2 and S3/D2 separately; and also of joined data sets, (S2 + S3), (S2 + S2/D2), (S2 + S3/D2), (S3 + S2/D2), (S3 + S3/D2) and (S2/D2 + S3/D2). Finally, we did the same calculations for the different filters data sets separately, AL1.0 and AL0.5, and also joining all the replicates (AL1.0 + AL0.5). The distance repeatabilities for separated data sets indicate the within-method reliability of measuring the skulls, whereas the repeatabilities for joined data sets comprise both within and between-method reliability.

### Raw and absolute differences within and between methods

In addition to the repeatability analysis, we also calculated raw and absolute differences of the distances between replicates, within and between methods. In the first case, we just subtracted the distances between replicates of the same individual and calculated the mean raw and absolute differences across all individuals for each distance. These calculations were made for each data set separately: MED, HIGH, DIG, S2, S3, S2/D2, S3/D3, AL1.0 and AL0.5. The between-method analysis was done by subtracting the distances between the same individuals measured with different methods (*e.g*., in the case of MED-HIGH, distances from individual 1 of MED were subtracted from the distances of individual 1 of HIGH, and so on for all 20 individuals) and calculating the raw and absolute mean differences across all individuals for each distance. All the differences that involved DIG (MED-DIG and HIGH-DIG) had 16 (4 skulls were lost in the cleaning procedure), while the rest of the differences between methods had 20 individuals. The raw differences show whether any method presents a consistent bias (e.g. under or overestimating the distances) in the data. The absolute differences indicate the magnitude of the error within and between methods, independent if the errors in the distances are in one direction or another. To get an idea of the error in relation to the distances means, we calculated the percentage error by dividing the absolute mean differences by their corresponding means and multiplying by 100.

### Statistical analysis

Correlation tests were all done using Pearson product moment correlation and significance level of 0.05. All ANOVAs in the repeatability analysis, correlation tests and graphics were done in the R environment [23].

## Additional file

Additional file 1: Table A1. Within-methods mean individual landmark distance without scale. **Table A2.** Between methods mean raw differences (in mm) in all the distances for joined data sets of micro-CT resolutions and 3D digitizer and of different filter type. **Table A3.** Mean percentage error in all distances for joined data sets of micro-CT resolutions and 3D digitizer and for distinct filter types.

## Abbreviations

AL0.5: Aluminum filter 0.5 mm
AL1.0: Aluminum filter 1.0 mm
DIG: 3 D digitizer
HIGH: High resolution
MED: Medium resolution
D2: Down-sample = 2.0
S2: Stride = 2.0
S3: Stride = 3.0
s^2^: Within-group variance
s^2^_A_: Among-group variance

## References

[1] Costantini D, Alonso ML, Moazen M, Bruner E (2010). The Relationship Between Cephalic Scales and Bones in Lizards: A Preliminary Microtomographic Survey on Three Lacertid Species. Anat Rec Adv Integr Anat Evol Biol.

[2] Ekdale EG (2010). Ontogenetic Variation in the Bony Labyrinth of Monodelphis domestica (Mammalia: Marsupialia) Following Ossification of the Inner Ear Cavities. Anat Rec Adv Integr Anat Evol Biol.

[3] Wilkinson M, San Mauro D, Sherratt E, Gower DJ (2011). A nine-family classification of caecilians (Amphibia: Gymnophiona). Zootaxa.

[4] Cuff AR, Rayfield EJ (2013). Feeding Mechanics in Spinosaurid Theropods and Extant Crocodilians. PLoS One.

[5] Gignac PM, Kley NJ (2014). Iodine-enhanced micro-CT imaging: Methodological refinements for the study of the soft-tissue anatomy of post-embryonic vertebrates. J Exp Zool B Mol Dev Evol.

[6] Rosset A, Spadola L, Ratib O (2004). OsiriX: An Open-Source Software for Navigating in Multidimensional DICOM Images. J Digit Imaging.

[7] Schunke AC, Bromiley PA, Tautz D, Thacker NA (2012). TINA manual landmarking tool: software for the precise digitization of 3D landmarks. Front Zool.

[8] Kohn LA, Cheverud JM (1992). Issues in evaluating repeatability of an imaging system for use in anthropometry.

[9] Kim G, Jung H-J, Lee H-J, Lee J-S, Koo S, Chang S-H (2012). Accuracy and Reliability of Length Measurements on Three-Dimensional Computed Tomography Using Open-Source OsiriX Software. J Digit Imaging.

[10] Halperin-Sternfeld M, Machtei E, Horwitz J (2014). Diagnostic Accuracy of Cone Beam Computed Tomography for Dimensional Linear Measurements in the Mandible. Int J Oral Maxillofac Implants.

[11] Zelditch ML (1988). Ontogenetic variation in patterns of phenotypic integration in the laboratory rat. Evolution.

[12] Cheverud JM (1995). Morphological Integration in the Saddle-back tamarin (Saguinus fuscicollis) cranium. Am Nat.

[13] Young NM, Hallgrímsson B (2005). Serial homology and the evolution of mammalian limb covariation structure. Evolution.

[14] Porto A, de Oliveira FB, Shirai LT, De Conto V, Marroig G (2009). The Evolution of Modularity in the Mammalian Skull I: Morphological Integration Patterns and Magnitudes. Evol Biol.

[15] Corner BD, Lele S, Richtsmeier JT (1992). Measuring Precision of Three-Dimensional Landmark Data. J Quant Antrophology.

[16] Richtsmeier JT, Paik CH, Elfert PC, Cole TM, Dahlman HR (1995). Precision, Repeatability and Validation of the Localization of Cranial Landmarks Using Computed Tomography Scans. Cleft Palate Craniofac J.

[17] Zeldicth ML, Swiderski DL, Sheets HD, Fink WL (2004). Geometric Morphometrics for Biologists: A Primer.

[18] Richard AH, Parks CL, Monson KL (2014). Accuracy of standard craniometric measurements using multiple data formats. Forensic Sci Int.

[19] Stull KE, Tise ML, Ali Z, Fowler DR (2014). Accuracy and Reliability of measurements obtained from computed tomography 3D volume rendered images. Forensic Sci Int.

[20] Lessells CM, Boag PT (1987). Unrepeatable Repeatabilities: A Common Mistake. Auk.

[21] Fernandes, TMF, Adamczyk, J, Poleti, ML, Henriques, JFC, Friedland, B, Garib, DG: Comparison between 3D volumetric rendering and multiplanar slices on the reliability on linear measurements on CBCT images: an in vitro study. Journal of Applied Oral Science 2014. 10.1590/1678-775720130445 PMC4349120 25004053

[22] SkyScan (2011). SkyScan 1176. In vivo X-Ray Microtomograph Instruction Manual.

[23] Bromiley PA, Ragheb H, Thacker NA (2012). The TINA Morphometrics Geometric Toolkit.

[24] R Core Team: R: A language and environment for statistical computing. R Foundation for Statistical Computing, Vienna, Austria 2013. URL: http://www.R-project.org/.

[25] Bookstein, FL: Morphometrics tools for landmark data: Geometry and Biology. Cambridge University Press. 1997. p. 425.

[26] Lele S (1993). Euclidean distance matrix analysis (EDMA): Estimation of mean form and mean form difference. Math Geol.

[27] van der Linde K, Houle D (2009). Inferring the Nature fo Allometry from Geometric Data. Evol Biol.

[28] Adams DC, Otarola-Castillo E (2013). geomorph: an R package for the collection and analysis of geometric morphometric shape data. Methods Ecol Evol.

[29] Dryden, IL: shapes: Statistical Shape Analysis. URL: http://cran.r-project.org/web/packages/shapes/index.html.

