# Supplementary material for: Landmark precision and reliability and accuracy of linear distances estimated by using 3D computed micro-tomography and the open-source TINA Manual Landmarking Tool software
Source: Front Zool. 2015 Jun 3;12:12. doi: 10.1186/s12983-015-0101-5 (PMC4481120; doi:10.1186/s12983-015-0101-5)
Supplement: Additional file 1: Table A1. — Within-methods mean individual landmark distance without scale. Table A2. Between methods mean raw differences (in mm) in all the distances for joined data sets of micro-CT resolutions and 3D digitizer and of different filter type. Table A3. Mean percentage error in all distances for joined data sets of micro-CT resolutions and 3D digitizer and for distinct filter types. [file 12983_2015_101_MOESM1_ESM.pdf]

Additional material files

Table A1. Within-methods mean individual landmark distance without scale. The table shows the mean individual landmark distances from the mean landmark. We used Generalized Procrustes Analysis (GPA) to superimpose individual landmarks, but we avoided the spread of variation from any one landmark to the others (see text). The values refer to a shape space and do not have scale. Values in bold are the highest deviations for DIG compared to MED and HIGH.

**Mean**									
**Landmarks**	**DIG**	**HIGH**	**MED**	**AL1.0**	**AL0.5**	**S2**	**S2/D2**	**S3**	**S3/D2**
1	0.0135	0.0104	0.0100	0.0116	0.0095	0.0109	0.0106	0.0102	0.0111
2	0.0128	0.0097	0.0111	0.0072	0.0072	0.0110	0.0109	0.0100	0.0115
**3**	**0.0161**	0.0086	0.0083	0.0105	0.0095	0.0085	0.0089	0.0085	0.0080
4	0.0131	0.0093	0.0115	0.0090	0.0102	0.0125	0.0121	0.0125	0.0125
5	0.0164	0.0112	0.0106	0.0112	0.0182	0.0107	0.0111	0.0115	0.0106
**6**	**0.0165**	0.0085	0.0093	0.0111	0.0127	0.0098	0.0089	0.0092	0.0099
**7**	**0.0156**	0.0081	0.0082	0.0074	0.0064	0.0076	0.0078	0.0079	0.0075
8	0.0152	0.0147	0.0122	0.0125	0.0161	0.0130	0.0129	0.0139	0.0136
**9**	**0.0213**	0.0098	0.0100	0.0117	0.0124	0.0105	0.0096	0.0108	0.0098
**10**	**0.0172**	0.0083	0.0082	0.0082	0.0089	0.0081	0.0079	0.0084	0.0085
11	0.0137	0.0083	0.0087	0.0078	0.0069	0.0089	0.0085	0.0084	0.0069
12	0.0196	0.0129	0.0138	0.0113	0.0109	0.0137	0.0133	0.0133	0.0124
13	0.0154	0.0126	0.0136	0.0146	0.0164	0.0124	0.0121	0.0126	0.0128
**14**	**0.0226**	0.0103	0.0094	0.0083	0.0079	0.0091	0.0094	0.0102	0.0102
**15**	**0.0154**	0.0064	0.0069	0.0072	0.0086	0.0068	0.0065	0.0067	0.0064
**16**	**0.0114**	0.0063	0.0064	0.0070	0.0077	0.0077	0.0060	0.0068	0.0067
17	0.0120	0.0079	0.0092	0.0094	0.0074	0.0088	0.0087	0.0086	0.0091
18	0.0160	0.0103	0.0106	0.0086	0.0103	0.0104	0.0120	0.0110	0.0104
**19**	**0.0233**	0.0099	0.0115	0.0122	0.0123	0.0102	0.0100	0.0097	0.0100
**20**	**0.0217**	0.0090	0.0080	0.0096	0.0097	0.0089	0.0093	0.0089	0.0087

Table A2. Between methods mean raw differences (in mm) in all the distances for joined data sets of micro-CT resolutions and 3D digitizer and of different filter type. The table shows the mean raw differences and their associated standard deviations in the length of the linear distances taken in the skulls between the same individuals scanned at medium (MED) and high (HIGH) resolutions and measured with TINA-Landmark (MED-HIGH); between the same individuals scanned at MED and measured with a 3D digitizer (DIG; MED-DIG); between the same individuals scanned at HIGH and measured with DIG (HIGH-DIG) and between the same individuals scanned with different thickness of an aluminum filter (AL1.0-AL0.5). The mean length of each distance is also shown as well as its correspondence to skull bones. Bold values indicate differences above 0.17 mm in magnitude. Positive and negative values for joined data sets MED-DIG and HIGH-DIG are for distances that were overestimated and underestimated, respectively, by measuring with the micro-CT compared to the 3D digitizer.

			Between methods raw differences (mm)			
**Distances**	**Means (mm)**	**Bones**	**MED-HIGH**	**MED-DIG**	**HIGH-DIG**	**AL1.0-AL0.5**
**1**	**4.06**	**nasal**	-0.03 ± 0.15	**-0.29 ± 0.30**	**-0.26 ± 0.27**	-0.05 ± 0.12
**2**	7.76	frontoparietal	-0.02 ± 0.23	0.04 ± 0.34	0.06 ± 0.38	0.14 ± 0.63
**3**	**6.55**	**nasal**	0.08 ± 0.27	**0.33 ± 0.41**	**0.23 ± 0.37**	0.09 ± 0.21
**4**	5.47	nasal	-0.02 ± 0.14	-0.15 ± 0.28	-0.13 ± 0.25	0.02 ± 0.21
**5**	**2.24**	**frontoparietal**	0.0 ± 0.06	**0.22 ± 0.13**	**0.22 ± 0.13**	-0.08 ± 0.51
**6**	**4.49**	**frontoparietal**	-0.03 ± 0.12	**-0.18 ± 0.20**	-0.14 ± 0.21	-0.15 ± 0.53
**7**	**5.69**	**orbit**	-0.06 ± 0.12	**-0.26 ± 0.27**	**-0.19 ± 0.28**	-0.08 ± 0.14
**8**	**2.82**	**squamosal**	0.0 ± 0.07	**0.19 ± 0.14**	**0.19 ± 0.15**	-0.06 ± 0.11
**9**	**3.64**	**occipital**	0.02± 0.11	**0.23 ± 0.23**	**0.21 ± 0.22**	-0.03 ± 0.16
**10**	1.82	frontoparietal	-0.03 ± 0.11	-0.07 ± 0.21	-0.02 ± 0.19	0.03 ± 0.19
**11**	2.73	prenasal	0.04 ± 0.08	-0.03 ± 0.20	-0.08 ± 0.21	0.03 ± 0.13
**12**	4.52	nasal	-0.04 ± 0.11	0.01 ± 0.29	0.05 ± 0.25	0.02 ± 0.13
**13**	**2.64**	**nasal**	-0.09 ± 0.10	**0.27 ± 0.20**	**0.36 ± 0.19**	-0.06 ± 0.15
**14**	**3.87**	**nasal**	0.02 ± 0.11	**-0.24 ± 0.23**	**-0.27 ± 0.19**	-0.01 ± 0.35
**15**	**8.47**	**maxilla**	-0.07 ± 0.16	**-0.26 ± 0.34**	**-0.17 ± 0.27**	-0.07 ± 0.16
**16**	**5.51**	**squamosal**	-0.02 ± 0.16	**-0.19 ± 0.70**	**-0.18 ± 0.73**	-0.07 ± 0.19
**17**	**7.14**	**parasphenoid**	**-0.17 ± 0.27**	**-0.27 ± 0.45**	-0.06 ± 0.46	-0.10 ± 0.24
**18**	**5.56**	**parasphenoid**	**-0.18 ± 0.25**	**-0.38 ± 0.35**	-0.16 ± 0.36	-0.01 ± 0.19
**19**	1.60	premaxilla	0.02 ± 0.07	-0.08 ± 0.13	-0.10 ± 0.10	-0.08 ± 0.07
**20**	**4.37**	**nasal**	-0.01 ± 0.11	**0.26 ± 0.22**	**0.27 ± 0.24**	0.07 ± 0.09
**21**	**4.74**	**neopalatine**	-0.04 ± 0.08	**0.21 ± 0.16**	**0.25 ± 0.15**	0.03 ± 0.10
**22**	5.06	pterygoid	0.0 ± 0.15	0.01 ± 0.20	0.01 ± 0.17	-0.03 ± 0.08
**23**	**4.58**	**pterygoid**	-0.06 ± 0.27	0.13 ± 0.39	**0.20 ± 0.63**	-0.03 ± 0.09
**24**	2.79	parasphenoid	0.01 ± 0.12	-0.05 ± 0.36	-0.04 ± 0.36	-0.05 ± 0.14

Table A3. Mean percentage error in all distances for joined data sets of micro-CT resolutions and 3D digitizer and for distinct filter types. Mean percentage error were calculated from differences in the skull distances between: (1) individuals scanned with medium resolution and the same individuals scanned with high resolution (MED-HIGH); (2) individuals scanned with MED and with a 3D digitizer (MED-DIG); (3) individuals scanned with HIGH and DIG (HIGH-DIG) and (4) individuals scanned with an AL 1.0 mm filter and the same individuals scanned with a AL 0.5 mm filter (AL1.0-AL0.5). The second column are the distances' means in mm. Values on bold correspond to mean percentages around 10.0%.

		Between methods mean percentage error			
**Distances**	**Means** (mm)	**MED-HIGH** (%)	**MED-DIG** (%)	**HIGH-DIG** (%)	**AL1.0-AL0.5** (%)
**1**	4.06	2.86	7.45	6.04	2.35
**2**	7.76	1.94	3.00	3.66	3.49
**3**	6.55	3.35	6.75	5.39	2.57
**4**	5.47	1.93	3.91	3.18	2.19
**5**	**2.24**	2.30	**10.79**	**10.75**	7.35
**6**	4.49	1.97	4.39	3.80	5.20
**7**	5.69	1.85	4.96	4.14	2.11
**8**	2.82	1.89	7.75	7.93	2.93
**9**	3.64	2.23	8.15	7.76	3.81
**10**	1.82	4.57	8.88	7.99	7.28
**11**	2.73	2.50	5.45	6.23	3.87
**12**	4.52	1.92	4.14	3.88	1.89
**13**	**2.64**	4.00	**11.61**	**15.17**	3.93
**14**	3.87	2.24	5.85	6.42	4.53
**15**	8.47	1.71	3.67	2.77	1.26
**16**	**5.51**	2.26	**9.32**	**9.82**	2.67
**17**	7.14	2.88	5.31	4.34	2.34
**18**	5.56	3.71	7.11	5.36	2.48
**19**	1.60	3.65	7.23	6.61	6.54
**20**	4.37	1.56	7.36	7.72	1.81
**21**	4.74	1.43	5.11	5.77	1.55
**22**	5.06	1.54	2.67	2.61	1.14
**23**	4.58	2.76	6.16	7.80	1.59
**24**	**2.79**	3.25	**8.65**	**9.19**	4.04
